# Restored retinal physiology after administration of niacin with citicoline in a mouse model of hypertensive glaucoma

**DOI:** 10.3389/fmed.2023.1230941

**Published:** 2023-09-05

**Authors:** Alberto Melecchi, Rosario Amato, Massimo Dal Monte, Dario Rusciano, Paola Bagnoli, Maurizio Cammalleri

**Affiliations:** ^1^Department of Biology, University of Pisa, Pisa, Italy; ^2^Interdepartmental Research Center Nutrafood “Nutraceuticals and Food for Health”, University of Pisa, Pisa, Italy; ^3^Research Center, Fidia Farmaceutici S.p.A, Catania, Italy

**Keywords:** intraocular pressure, oxidative stress, inflammation, mitochondrial dysfunction, apoptotic cascade, electroretinography

## Abstract

**Introduction:**

Much interest has been addressed to antioxidant dietary supplements that are known to lower the risk of developing glaucoma or delay its progression. Among them, niacin and citicoline protect retinal ganglion cells (RGCs) from degeneration by targeting mitochondria, though at different levels. A well-established mouse model of RGC degeneration induced by experimental intraocular pressure (IOP) elevation was used to investigate whether a novel combination of niacin/citicoline has better efficacy over each single component in preserving RGC health in response to IOP increase.

**Methods:**

Ocular hypertension was induced by an intracameral injection of methylcellulose that clogs the trabecular meshwork. Electroretinography and immunohistochemistry were used to evaluate RGC function and density. Oxidative, inflammatory and apoptotic markers were evaluated by Western blot analysis.

**Results:**

The present results support an optimal efficacy of niacin with citicoline at their best dosage in preventing RGC loss. In fact, about 50% of RGCs were spared from death leading to improved electroretinographic responses to flash and pattern stimulation. Upregulated levels of oxidative stress and inflammatory markers were also consistently reduced by almost 50% after niacin with citicoline thus providing a significant strength to the validity of their combination.

**Conclusion:**

Niacin combined with citicoline is highly effective in restoring RGC physiology but its therapeutic potential needs to be further explored. In fact, the translation of the present compound to humans is limited by several factors including the mouse modeling, the higher doses of the supplements that are necessary to demonstrate their efficacy over a short follow up period and the scarce knowledge of their transport to the bloodstream and to the eventual target tissues in the eye.

## 1. Introduction

Glaucoma is a chronic optic neuropathy characterized by progressive degeneration of retinal ganglion cells (RGCs), which leads to progressive visual loss (1, 2). Elevated intraocular pressure (IOP) and the derived mechanical stress are classically considered as the main causes of RGC death. In fact, increased IOP leads to excessive production of reactive oxygen species (ROS) that plays a role in the pathogenesis of glaucoma stimulating apoptotic and inflammatory pathways and promoting RGC apoptosis. Lifestyle and dietary supplementation may influence some of the risk factors and pathophysiological mechanisms underlying glaucoma development and progression. Antioxidant, anti-inflammatory and neuroprotective properties of ingredients in the dietary regimen have shown promising results in the management of chronic degenerative ocular diseases including glaucoma. For instance, intervening on the chronic intake of active molecules such as caffeine has been recently reported to exert protective effects on RGCs through anti-inflammatory action (3). In addition, impairment of mitochondrial function by mechanical stress and decreased blood perfusion due to IOP increase is likely to affect RGC survival thus eliciting a great interest on strategies that intervene on mitochondrial dysfunction in order to delay or even halt RGC loss (4–6). Some dietary supplements targeting mitochondrial dysfunction have been demonstrated to lower the risk of developing glaucoma and potentially slow disease progression. Among them, niacin (vitamin B3 or nicotinamide, a precursor of nicotinamide adenine dinucleotide, NAD^+^) is largely present in healthy diets while citicoline (Cytidine 5′-diphosphocholine), an intermediate in the generation of phosphatidylcholine from choline, is found in small amount in only a few food groups. Although acting at different cellular levels, both compounds represent to date some of the most promising neuroprotective supplements in ophthalmology with growing evidence demonstrating their efficacy against RGC degeneration (7–10). In particular, niacin acts as a crucial regulator of mitochondrial metabolism and redox reactions (11) while citicoline stabilizes cell membranes through the synthesis of phospholipids (12) and the production of cardiolipin, thus inhibiting mitochondria-mediated apoptosis (13). In glaucoma models, diets supplemented with niacin have been demonstrated to protect RGCs from degeneration by supporting mitochondrial health and metabolism (14–16). In this respect, vitamin B3 has been included in the panel of neuroprotective strategies in glaucoma patients in which low vitamin B3 levels have been demonstrated (17). Accordingly, vitamin B3 supplementation appears to counteract RGC dysfunction. In fact, in humans, increased niacin intake is associated with a lower likelihood of glaucoma (18) and NAD precursors seem to reduce the RGC vulnerability to increased IOP (19). In addition to niacin, citicoline protection against RGC damage has been extensively demonstrated in different models of RGC injury (20, 21). At the clinical level, citicoline administration to glaucoma patients appears to slow down glaucoma progression by exerting neuroprotective effects (22, 23). Preclinical and clinical data on the efficacy of citicoline supplementation in glaucoma have been comprehensively reviewed by Grieb et al. (24). The fact that glaucomatous degeneration encompasses multiple molecular pathways with intricate interactions suggests an important role of multitarget approaches based on the use of agent combinations acting on complementary pathogenic mechanisms. For in-stance, oxidative stress leads to dysfunctional mitochondria, which in turn may modulate inflammatory processes, while pro-inflammatory mediators also alter mitochondrial function (25). In fact, the excess of reactive oxygen species (ROS) not compensated by endogenous antioxidant defenses can damage lipids, DNA, and proteins leading to RGC degeneration and can also activate the release of inflammatory mediators that participate to ROS-mediated RGC apoptosis (26). To this end, dietary supplements have been recognized as a promising adjuvant therapy in glaucoma since they may counteract oxidative stress and mitochondrial dysfunction (27, 28). The fact that both niacin and citicoline tar-get mitochondria made us to hypothesize that their association could be more effective than the action of each single molecule. Therefore, aim of this study has been the evaluation of the efficacy of a dietary supplementation with niacin and citicoline in a mouse model of ocular hypertension induced by the injection of 2% methylcellulose (MCE) into the anterior chamber (29, 30). In this model, the efficacy of niacin/citicoline, either alone or in association, has been determined on the pathological hallmarks leading to RGC death – including oxidative stress and inflammation – both converging on the apoptotic cascade that culminates in RGC loss. Further evidence on the combination efficacy on RGCs survival have been also provided by the electrophysiological assessment of RGC function (29–31).

## 2. Materials and methods

### 2.1. Animals

C57BL/6 J male mice (2-month-old) were purchased from Charles River Laboratories Italy (Calco, Italy). Mice were housed in a constant environment (23 ± 1°C, 50 ± 5% humidity) with a 12 h light/dark cycle (lights on at 08.00 a.m.) and fed with a standard diet and water *ad libitum*. Before starting the study, all mice were acclimatized for 7 days to handling and tonometry. This study was carried out in compliance with the ARVO Statement for the Use of Animals in Ophthalmic and Vision Research. The present study follows the European Communities Council Directive (2010/63/UE) and the Italian guidelines for animal care (DL 26/14, permission number: 132/2019PR). The 3Rs principles for ethical use of animals in scientific research guided our efforts to reduce both the number and suffering of the animals used in the present study. A total of 60 mice with an average body weight of 20–25 g were randomly assigned to 10 different groups as reported in Table 1.

**Table 1:** Experimental groups.

Experimental group	MCE	Treatment	Sample size
Control		Water	6
MCE	MCE injection	Water	6
N low	MCE injection	Niacin 0.5 g/Kg/die	6
C low	MCE injection	Citicoline 0.5 g/Kg/die	6
N high	MCE injection	Niacin 2.5 g/Kg/die	6
C high	MCE injection	Citicoline 1 g/Kg/die	6
N low + C low	MCE injection	Niacin 0.5 g/Kg/die + citicoline 0.5 g/Kg/die	6
N high + C low	MCE injection	Niacin 2.5 g/Kg/die + citicoline 0.5 g/Kg/die	6
N low + C high	MCE injection	Niacin 0.5 g/Kg/die + citicoline 1 g/Kg/die	6

N: niacin; C: citicoline.

### 2.2. Experimental model of ocular hypertension

Ocular hypertension was induced by an intracameral injection of 2% MCE in agreement with previous studies (29, 30). MCE (M0512; Sigma Aldrich, St. Louis, MO, United States) was dissolved at 2% in sterile saline to obtain an aqueous solution with a viscosity ranging from 3,500 to 5,600 cps. Mice were anesthetized with an intraperitoneal injection of avertin (1.2% tribromoethanol and 2.4% amylene hydrate in distilled water, 0.02 mL/g body weight: Sigma-Aldrich) and injected into the anterior chamber with 5 μL of MCE in both eyes by means of a Hamilton syringe equipped with a 36-gauge needle. The needle was inserted in the iridocorneal angle at about 1 mm from ora serrata and oriented parallelly to the iris surface. After MCE injections, mice were daily monitored for any alteration in ocular tissues (e.g., cataract, corneal opacity or edema).

### 2.3. Oral supplementation with niacin and citicoline or their combination

Niacin and citicoline powders were supplied by Fidia Farmaceutici (Abano Terme, PD, Italy) and dissolved in drinking water either alone or in association. In light of pre-liminary observations showing an average daily drinking volume of 5 mL/mouse/day and assuming an average mouse body weight of 20 g, niacin was dissolved at low (2 mg/mL) or high concentration (10 mg/mL) in order to obtain a corresponding daily dosage of 0.5 g/Kg (low dose; N low) or 2.5 g/Kg (high dose; N high). Similarly, citicoline was dissolved in the drinking water at low (2 mg/mL) or high concentration (4 mg/mL) to obtain a corresponding daily dosage of 0.5 g/Kg (low dose; C low) or 1 g/Kg (high dose; C high). Niacin and citicoline doses used here are in line with those reported in previous studies (21, 32, 33). Treatments providing different dose combinations of the two components were obtained by dissolving both niacin and citicoline in the same solution at the corresponding concentration. Mice were treated 14 days before and 14 days after the induction of ocular hypertension.

### 2.4. IOP measurement

IOP was non-invasively assessed every day using rebound tonometry (TonoLab, Icare Finland Oy, Helsinki, Finland). Mice were gently restrained, with the probe of the tonometer aligned to the eye optical axis at a 1–2 mm distance from the cornea. After habituation, the average of 5 consecutive recordings was considered as a reliable measure of IOP.

### 2.5. Electroretinogram

Full field photopic ERG was recorded in control mice and in mice that received MCE, untreated or treated with niacin and citicoline either alone or in combination. Mice dark-adapted overnight were anesthetized with intraperitoneal injection of avertin and placed on a heating pad. The body temperature was maintained at 38°C using a homeo-thermic controller. To maintain dark adaptation all handling and experiments were performed under a dim red light. ERG responses were recorded using Ag/AgCl corneal electrodes. A reference electrode was inserted on the forehead, while a ground electrode was inserted at the base of the tail. The cornea was intermittently irrigated with saline solution to prevent clouding of the ocular media. Electroretinographic recordings were made using an ERG setup (Retimax Advanced, CSO, Firenze, Italy). Following light-adaptation (10 min to 30 cd-s/m^2^) to suppress the rod response, photopic responses were obtained following the delivery of 10 consecutive stimuli at 3 cd-s/m^2^ with an interstimulus interval of 3 s and averaged to minimize the noise contribution. In the photopic ERG, the photopic negative response (PhNR) was measured from the baseline (0 μV) to the trough of the negative response following the positive b-wave.

PERG responses were evoked by delivering visual stimuli consisting of black-white horizontal bars (0.05 cycles/deg. black and white bars reversing at 1 Hz presented at 98% contrast) generated on a light emitting diode display with a mean luminance of 50 cd/m^2^ aligned at about 25 cm from the cornea surface. A total of 300 consecutive signals was averaged to reduce noise contamination. PERG responses were analyzed by measuring the N35-P50 amplitude (from the negative peak, N35, to the positive peak, P50) and the P50-N95 amplitude (from the positive peak, P50, to the negative peak, N95).

### 2.6. Retinal ganglion cell immunohistochemistry

After ERG recording, mice were sacrificed by an overdose of avertin and their eyeballs enucleated. Then, 6 random retinas were isolated and fixed in in 4% paraformaldehyde in 0.1 M phosphate buffered saline (PBS) for 2 h at room temperature. Contralateral retinas were used for molecular analyses. Retinas were rinsed with PBS and incubated for 24 h at 4°C with the primary antibody directed to RBPMS (ABN1376, Merck, Darmstadt, Germany) diluted 1:100 in PBS containing 2% TritonX-100 and 5% BSA. Retinas were then rinsed with PBS and incubated for 2 h with FITC-conjugated anti-guinea pig secondary antibody (F6261, Merck) diluted 1:200 in PBS containing 5% BSA and 2% TritonX-100. Finally, retinas were rinsed in PBS, flat mounted on polarized glass slides with the RGC layer facing up and coverslipped with a mounting medium. Images were acquired using an epifluorescence microscope (Ni-E; Nikon-Europe, Amsterdam, Netherlands) equipped with a digital camera (DS-Fi1c camera; Nikon-Europe). The number of RBPMS immuno-positive RGCs (number of cells per mm^2^) was compared between the experimental groups. Quantification was performed in a masked manner, with the operator ignoring the treatment received by the donor animal.

### 2.7. Western blot

Isolated retinas were homogenized in RIPA lysis buffer (Santa Cruz Biotechnology, Dallas, TX, Unites States) containing a cocktail of protease and phosphatase inhibitors (Roche Ap-plied Science, Indianapolis, IN, Unites States). Protein concentration of retinal homogenates was quantified by Micro BCA Protein Assay (Thermo Fisher Scientific, Waltham, MA, United States). Thirty micrograms of proteins from each sample were run on 4–20% SDS-PAGE gels (Bio-Rad Laboratories, Inc., Hercules, CA, Unites States) before transferring samples onto polyvinylidene difluoride membranes. Membranes were blocked for 1 h with 5% skim milk and then incubated overnight at 4°C with the solutions of primary antibodies listed in Table 2 using β-actin as the loading control. Membranes were then incubated for 2 h with rabbit polyclonal anti-mouse HRP-conjugated (A9044, Sigma-Aldrich) or goat polyclonal anti-rabbit HRP-conjugated (170–6,515, Bio-Rad Laboratories, Inc.) secondary antibodies (1:5000) and developed with the Clarity Western enhanced chemiluminescence substrate (Bio-Rad Laboratories, Inc.). Images were acquired using the ChemiDoc XRS+ (Bio-Rad Laboratories, Inc.). The optical density (OD) of the bands was evaluated using the Image Lab 3.0 software (Bio-Rad Laboratories, Inc.) and data were normalized to the corresponding OD of β-actin or nuclear factor kappa-light-chain-enhancer of activated B cells (NF-kB) p65 as appropriate.

**Table 2:** Western blot antibodies.

Antibodies	Dilution	Source	Catalogue
Rabbit polyclonal anti-Nrf-2	1:1000	Abcam (Cambridge, UK)	ab92946
Rabbit polyclonal anti-HO-1	1:500	Abcam	ab13243
Rabbit monoclonal anti-pNF-kB p65 (Ser 536)	1:1000	Abcam	ab76302
Rabbit polyclonal anti-NF-kB p65	1:1000	Abcam	ab16502
Mouse monoclonal anti-IL-6	1:500	Santa Cruz Biotech	sc-57,315
Rabbit monoclonal anti-GFAP	1:5000	Abcam	ab207165
Rabbit monoclonal anti-cleaved caspase 3	1:1000	Cell Signaling Technology (Danvers, MA, United States)	9,664
Rabbit monoclonal anti-Bax	1:500	Abcam	ab182733
Rabbit polyclonal anti-Bcl-2	1:500	Abcam	ab194583
Mouse monoclonal anti-cytochrome c	1:250	BD Biosciences (San Diego, CA, United States)	556,433
Mouse monoclonal anti-β-actin	1:2500	Sigma-Aldrich	A2228

### 2.8. Statistical analysis

Data were analyzed by the Shapiro–Wilk test to verify their normal distribution. Statistical analysis was carried out with Graph Pad Prism 8.0.2 software (GraphPad Soft-ware, Inc., San Diego, CA, United States). Multiple comparisons among groups were analyzed by two-way ANOVA followed by the Tukey *post hoc* test. Data are expressed as mean ± SEM of the indicated n values. Differences with *p* < 0.05 were considered as significant.

## 3. Results

### 3.1. Effect of dietary supplementation with niacin and citicoline on MCE-induced IOP elevation

**Figure 1:**
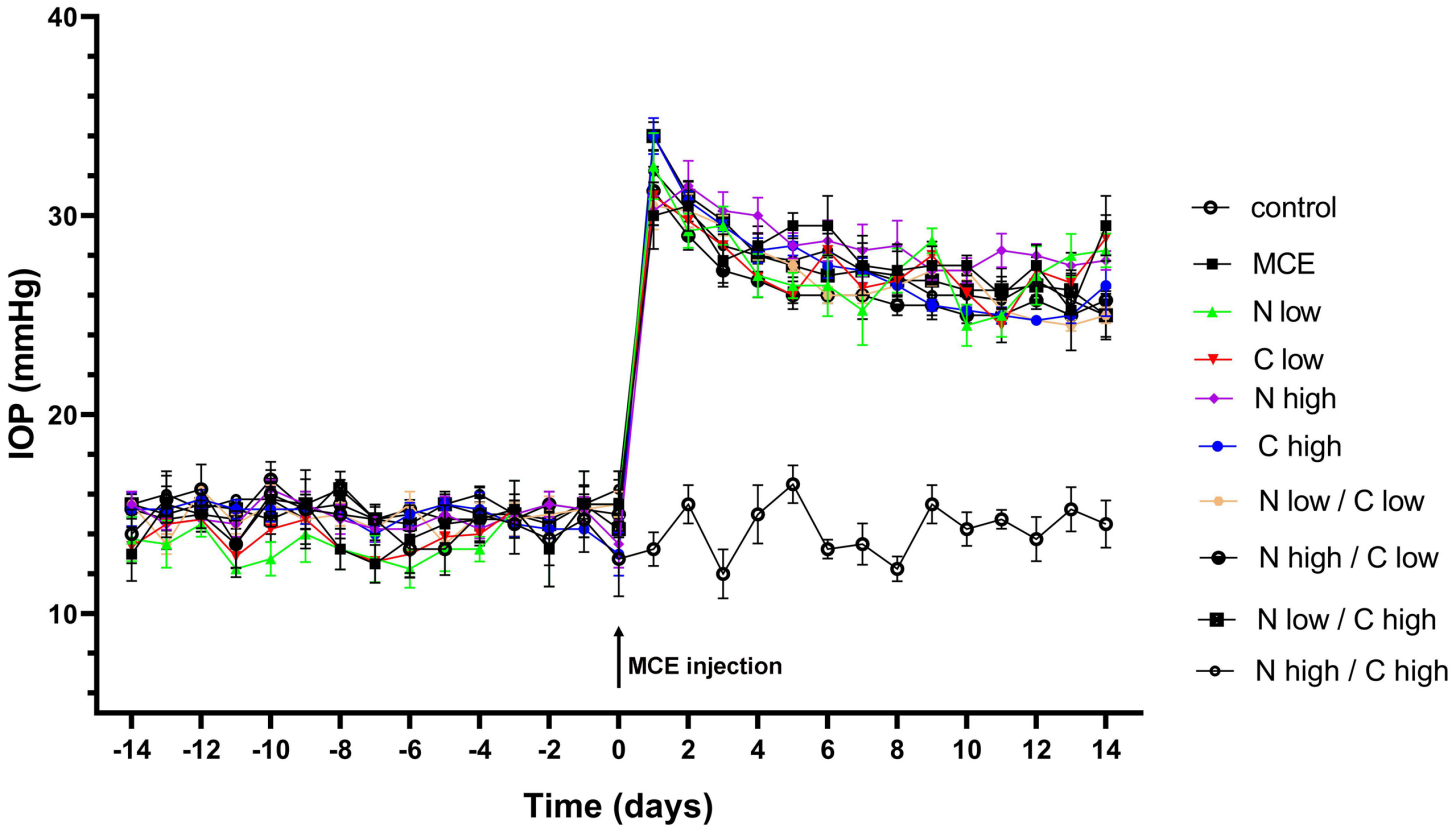
Effect of individual or combined administration of niacin and citicoline on methylcellulose (MCE)-induced ocular hypertension. Longitudinal evaluation of intraocular pressure (IOP) levels as assessed by rebound tonometry in control and MCE injected mice either untreated or treated with niacin and citicoline (individual or combined). Data are expressed as mean ± SEM of *n* = 6 mice.

IOP levels were determined in both untreated and treated mice before and after MCE injection. As shown in Figure 1, during the 14 days preceding MCE injection, IOP levels were stable at about 14 mmHg with no difference between treated and untreated groups, while 24 h after the injection, IOP levels significantly increased to reach a peak at about 32 mmHg. MCE-induced ocular hypertension was maintained up to 14 days in agreement with previous findings (29, 30). Either individual or combined administration of niacin and citicoline did not influence the MCE-induced IOP elevation.

### 3.2. Effect of dietary supplementation with niacin and citicoline on MCE-induced RGC dysfunctional activity

**Figure 2:**
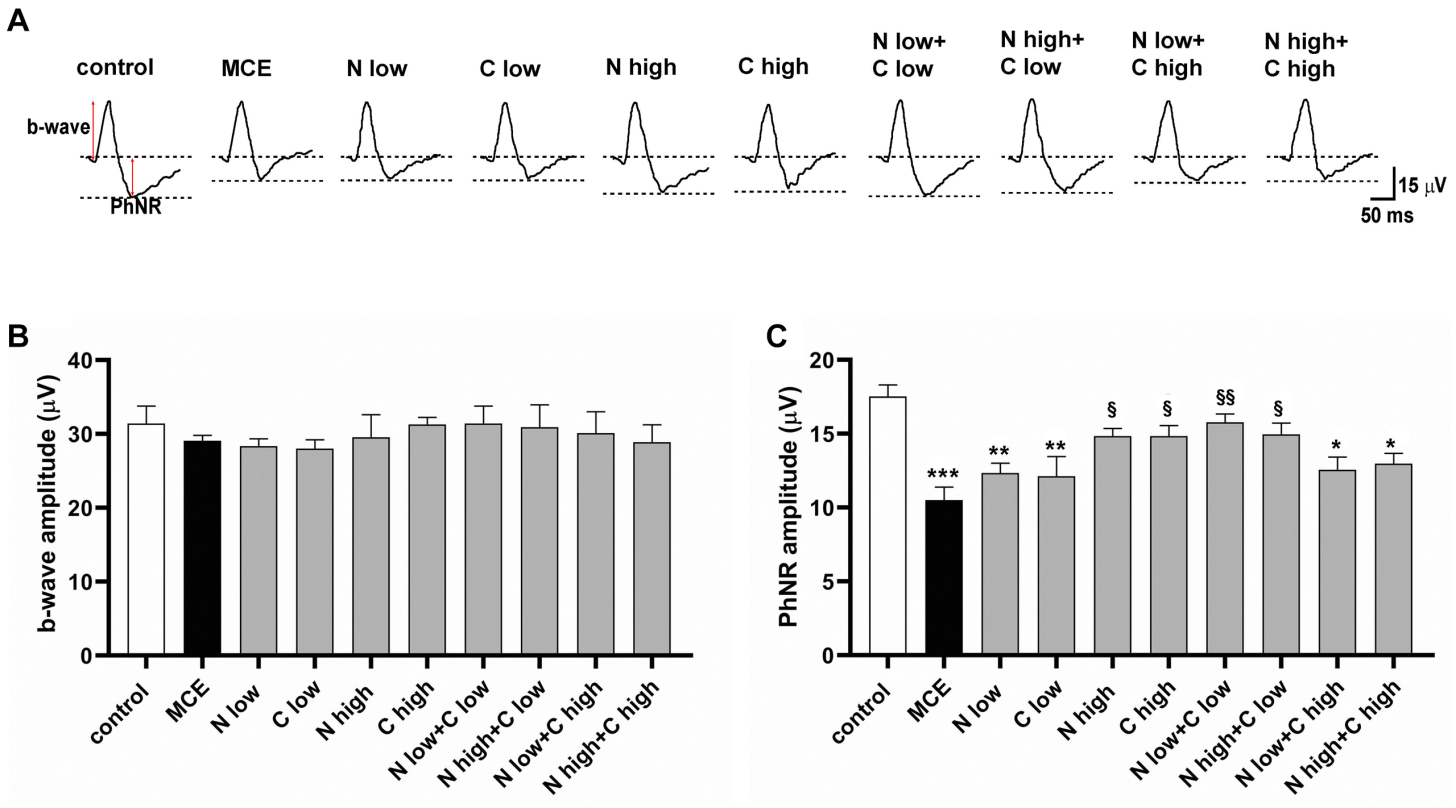
Effect of individual or combined administration of niacin with citicoline on photopic electro-retinogram (ERG). **(A)** Representative waveforms of photopic ERG in control and methylcellulose (MCE)-injected mice either untreated or treated with individual niacin and citicoline or their combination. **(B)** Mean amplitude of photopic b-wave. **(C)** Mean amplitude of photopic negative response (PhNR) as measured from the baseline (0 μV) to the trough of the negative response following the positive b-wave. Data are expressed as mean ± SEM (*n* = 6 mice for each experimental group). ^*^p < 0.01, ^**^p < 0.001, and ^***^p < 0.0001 vs. control; ^§^*p* < 0.01, ^§§^*p* < 0.001 vs. MCE (two-way ANOVA followed by Tukey’s multiple comparison post-hoc test).

The effect of niacin and citicoline administration, either alone or in combination, on MCE-induced retinal dysfunction was assessed by photopic ERG recordings, including the b-wave as a cone-driven post-receptor response and the photopic negative response (PhNR) as a RGC-related response (Figure 2A). In MCE-injected mice, the amplitude of the photopic b-wave did not display any statistical difference from the control group independent on treatment (Figure 2B). Conversely, PhNR amplitude in untreated mice receiving MCE injection, was significantly decreased by about 42% as compared to controls. Mice receiving single treatments with low dose of either niacin or citicoline displayed a PhNR amplitude comparable to that of MCE untreated mice. In contrast, single treatments with niacin or citicoline at high dose prevented the MCE-induced decrease in PhNR responses. When used in combination, either low or high dose of niacin preserved the PhNR amplitude only in the presence of citicoline at low dose, while the PhNR amplitude remained rather similar to that of MCE untreated mice in the presence of citicoline at high dose (Figure 2C).

**Figure 3:**
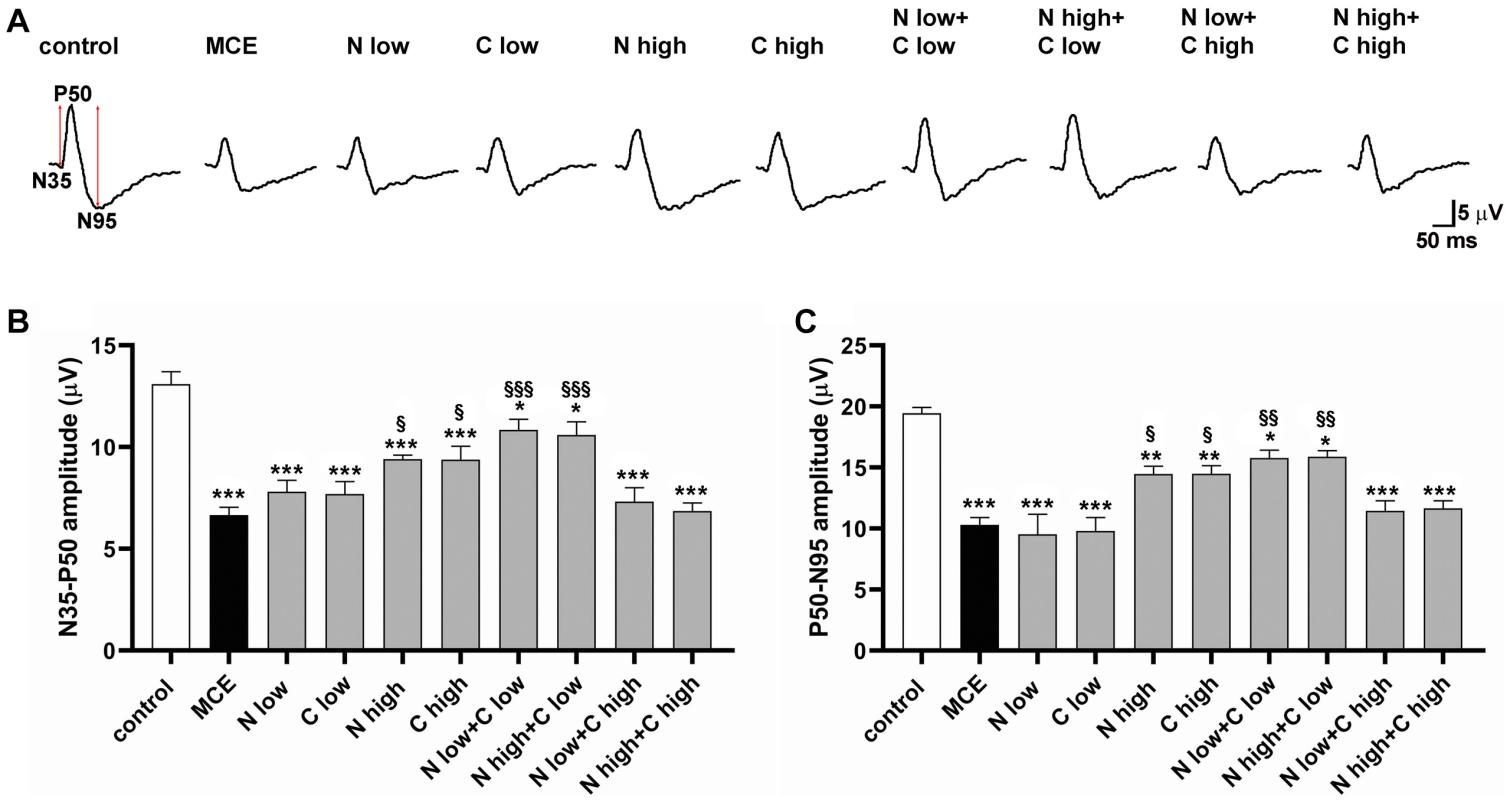
Effect of individual or combined administration of niacin and citicoline on methylcellulose (MCE)-induced dysfunctional pattern electroretinogram (PERG). **(A)** Representative waveforms of PERG in control and MCE-injected mice either untreated or treated with individual niacin and citicoline or their combination. **(B,C)** Mean amplitudes of the N35-P50 **(B)** and P50-N95 **(C)** waves. Data are expressed as mean ± SEM (*n* = 6 mice for each experimental group). ^*^p < 0.05, ^**^p < 0.001, and ^***^p < 0.0001 vs. control; ^§^*p* < 0.01, ^§§^*p* < 0.001, ^§§§^*p* < 0.0001 vs. MCE (two-way ANOVA followed by Tukey’s multiple comparison post-hoc test).

The RGC-specific activity in each experimental group was assessed by analyzing both the positive (N35-P50) and the negative components (P50-N95) of pattern ERG (PERG) responses (Figure 3A). MCE-injected mice showed a significant decrease in both N35-P35 and P50-N95 amplitudes as compared to controls (50% and 53%, respectively), without any significant effect exerted by individual treatments with either niacin or citicoline at low dose. On the contrary, the individual treatment with niacin or citicoline at high dose partially prevented the MCE-induced decrease in PERG responses, which still resulted about 25% lower than in controls. Both N35-P35 and P50-N95 amplitudes were preserved to control levels following the combined administration of niacin (at either low or high dose) with citicoline at low dose. In contrast, combined administration of citicoline at high dose did not influence PERG response amplitudes, which were comparable to those of MCE untreated mice (Figures 3B,C).

### 3.3. Effect of dietary supplementation with niacin and citicoline on MCE-induced RGC loss

**Figure 4:**
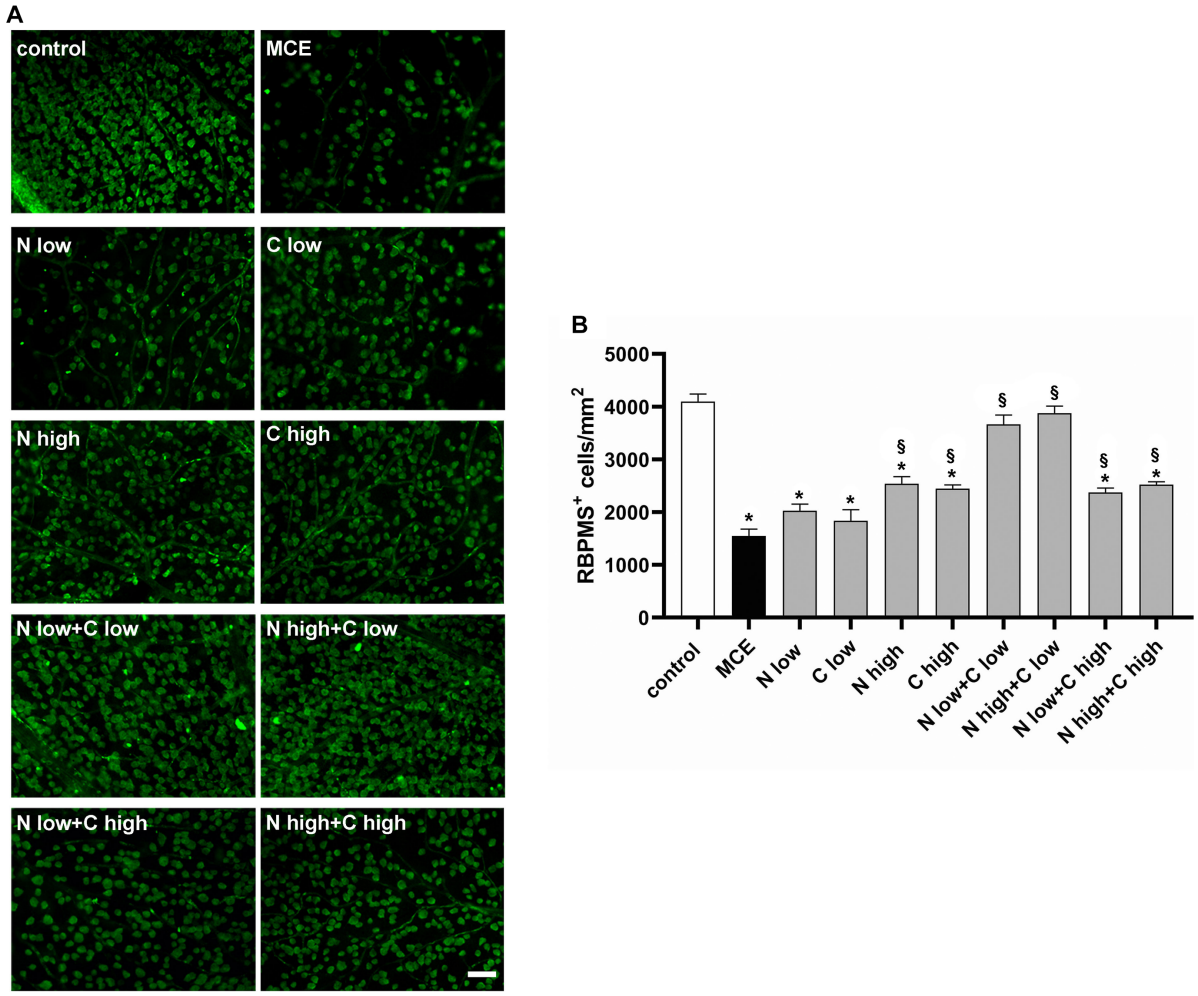
Effect of individual or combined administration of niacin and citicoline on methylcellulose (MCE)-induced retinal ganglion cell (RGC) loss. **(A)** Representative images of RNA-binding protein with multiple splicing (RBPMS) positive RGCs in whole-mount retinas of control and MCE mice either untreated or treated with individual niacin and citicoline or their combination. Scale bar: 50 μm. **(B)** Quantitative analysis of RBPMS-positive cell density. Data are expressed as mean ± SEM (*n* = 6 retinas for group). ^*^p < 0.0001 vs. control; ^§^*p* < 0.0001 vs. MCE (two-way ANOVA followed by Tukey’s multiple comparison post-hoc test).

Together with RGC dysfunction, the progressive decrease in RGC density represents a hallmark of MCE-induced glaucomatous damage. After recording RGC activity, we examined whether the improved retinal function after niacin (at either doses) and citicoline at low dose was accompanied by a reduced RGC loss by evaluating the density of RNA-binding protein with multiple splicing (RBPMS) positive RGCs in retinal flat mounts. As shown by the representative images in Figure 4A and their related quantification (Figure 4B), after MCE injection RGC density decreased by about 63% as compared to controls with no significant effects of the individual administration of niacin or citicoline at low dose. In mice receiving niacin or citicoline alone at high dose, RGC density was higher than in MCE untreated mice, but still 39% lower than in controls. The combined administration of niacin (at either doses) and citicoline at low dose preserved the RGC density to control levels. Both doses of niacin in combination with citicoline at high dose was found to slightly increase the RGC density that remained about 40% lower than in controls.

### 3.4. Effect of dietary supplementation with niacin and citicoline on oxidative stress and inflammatory markers

**Figure 5:**
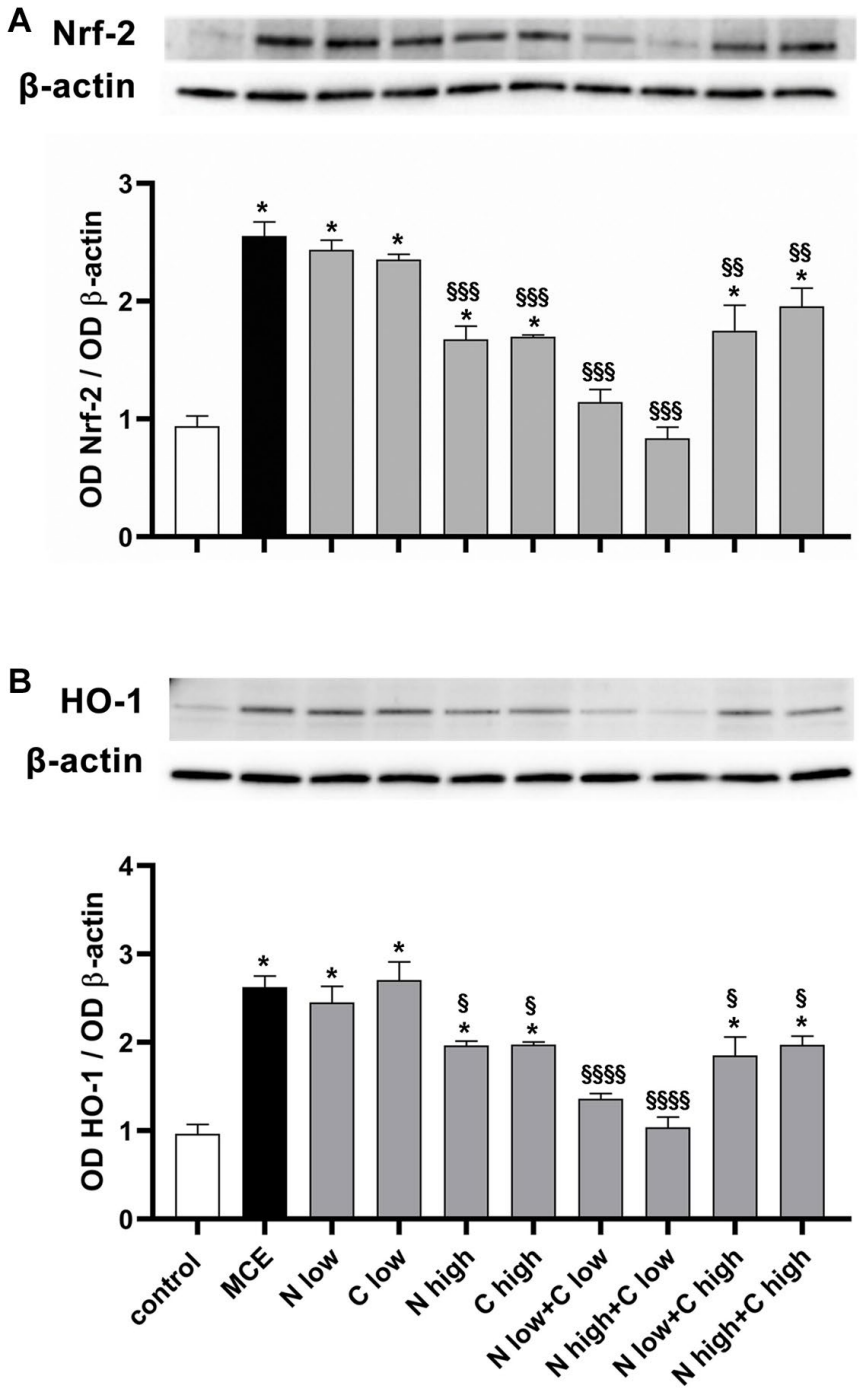
Effect of individual or combined administration of niacin and citicoline on MCE-induced oxidative stress. **(A)** Representative Western blots and densitometric analysis of nuclear factor erythroid 2-related factor-2 (Nrf-2) and **(B)** heme oxygenase-1 (HO-1) levels in control and MCE mice either untreated or treated with individual niacin and citicoline or their combination. Data are expressed as mean ± SEM (*n* = 6 retinas for group). ^*^p < 0.0001 vs. control; ^§^*p* < 0.01, ^§§^*p* < 0.001, and ^§§§^*p* < 0.0001 vs. MCE (two-way ANOVA followed by Tukey’s multiple comparison post-hoc test).

In the glaucomatous damage to RGCs, oxidative stress and inflammation represent primary events driving the progressive loss of RGC activity and viability. Retinal levels of typical markers of oxidative stress and inflammation were assessed following the individual or combined administration of niacin and citicoline to MCE-injected mice. In Figure 5, oxidative stress was evaluated by measuring protein levels of nuclear factor erythroid 2-related factor-2 (Nrf-2), a ROS-sensitive transcription factor, and the antioxidant enzyme heme oxygenase-1 [HO-1; (34)]. Protein levels of both Nrf-2 and HO-1 in-creased in MCE-untreated mice by about 270% and 272%, respectively, as compared with controls. Protein levels of both markers did not differ from those of MCE untreated mice after the administration of niacin or citicoline at low dose, while they were significantly decreased following the treatment with high dose of niacin (Nrf-2: −35%, HO-1: −25%) or citicoline (Nrf-2: −34%, HO-1: −25%) as compared to MCE untreated mice. Combined administration of niacin (at either low or hi gh dose) with citicoline at low dose further decreased oxidative stress markers to levels comparable to those in controls, whereas the combination of niacin (at either low or high dose) with citicoline at high dose only partially affected oxidative stress markers (Nrf-2: about −24%, HO-1: about −27%) as compared to MCE untreated mice.

**Figure 6:**
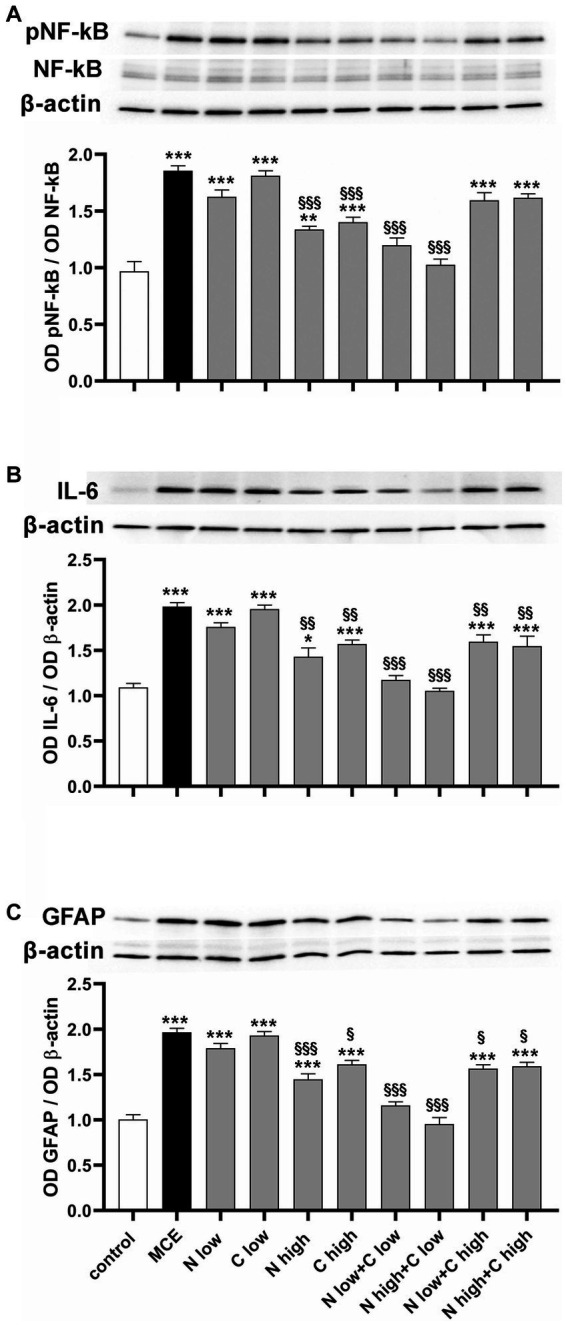
Effect of individual or combined administration of niacin and citicoline on MCE-induced in-flammatory response. **(A–C)** Representative Western blots and densitometric analysis of the phosphorylated form of the p65 subunit of nuclear factor kap-pa-light-chain-enhancer of activated B cells (pNF-kB) and NF-kB **(A)**, interleukin (IL)-6 **(B)** and glial fibrillary acidic protein (GFAP; **C**) levels in control MCE mice either untreated or treated with individual niacin and citicoline or their combination. Data are expressed as mean ± SEM (*n* = 6 retinas for group). ^*^p < 0.01, ^**^p < 0.001, and ^***^p < 0.0001 vs. control; ^§^*p* < 0.01, ^§§^*p* < 0.001, and ^§§§^*p* < 0.001 vs. MCE (two-way ANOVA followed by Tukey’s multiple comparison post-hoc test).

Inflammation processes and associated glial reactivity were evaluated by measuring the protein levels of the phosphorylated form of the p65 subunit of nuclear factor kappa-light-chain-enhancer of activated B cells (pNF-kB), a master transcriptional regulator of pro-inflammatory factors including interleukin (IL)-6 (35) and the glial fibrillary acid protein (GFAP) as a gliosis marker. In untreated mice injected with MCE, the protein level of pNF-kB was significantly increased by about 92% (Figure 6A) while the protein level of IL-6 increased by about 98% (Figure 6B). The individual administration of niacin or citicoline at low dose did not influence the MCE-induced increment in pNF-kB and IL-6, while their administration at high dose significantly reduced the levels of both inflammatory markers, although they remained still higher than in controls (+40% and + 45%, respectively). The combined administration of niacin with citicoline at low dose prevented the MCE-induced increase of pNF-kB and IL-6. The combination efficacy on pNF-kB increase was lost when citicoline was administered at high dose, while a slight decrement was observed on IL-6 protein levels (−19%). MCE-induced increase in GFAP protein levels (+95%) was significantly decreased by individual niacin and citicoline at high dose (−42% and −43%, respectively). Niacin (at either doses) in combination with citicoline at low dose preserved GFAP at levels similar to those in controls, while in combination with at high dose had lower efficacy on MCE-induced GFAP accumulation that remained about 55% higher than in controls (Figure 6C).

### 3.5. Effect of dietary supplementation with niacin and citicoline on intrinsic apoptotic pathway

**Figure 7:**
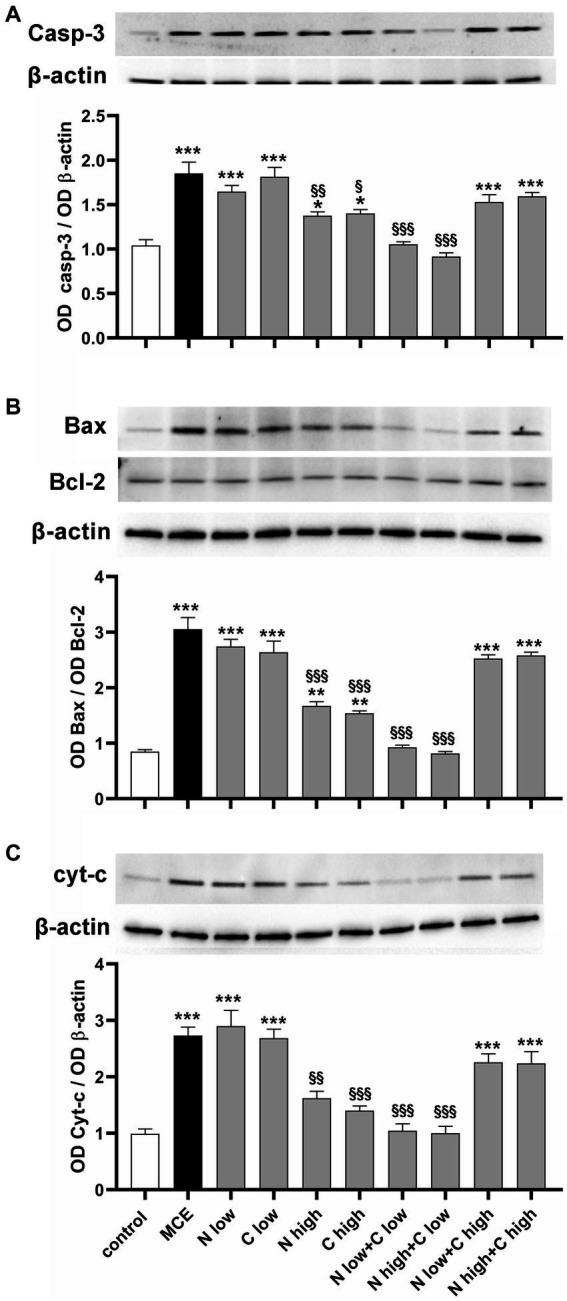
Effect of individual or combined administration of niacin and citicoline on MCE-induced apoptotic cascade. **(A–C)** Representative Western blots and densitometric analysis of caspase 3 **(A)**, Bax/Bcl-2 ratio **(B)** and cytochrome c **(C)** in control and MCE mice either untreated or treated with individual niacin and citicoline or their combination. Data are expressed as mean ± SEM (*n* = 6 retinas for group). ^*^p < 0.05, ^**^p < 0.001, and ^***^p < 0.0001 vs. control; ^§^*p* < 0.01, ^§§^*p* < 0.001 and ^§§§^*p* < 0.001 vs. MCE (two-way ANOVA followed by Tukey’s multiple comparison post-hoc test).

Altered oxidative stress and inflammatory processes lead to progressive RGC death by triggering the apoptotic cascade in which the final executioner is the active caspase-3. As shown in Figure 7A, in MCE untreated mice, the level of caspase-3 was increased with respect to controls (+84%). It was not significantly affected by the administration of niacin or citicoline at low dose while it was drastically reduced by their administration at high dose (about −45%). Combined administration of niacin (at either low or high dose) with citicoline at low dose further decreased caspase-3 protein levels which became comparable to those in controls. Conversely, combined treatment with citicoline at high dose did not affect caspase-3 levels that remained comparable to those in untreated MCE mice. To evaluate the combination efficacy on the mitochondrial-dependent intrinsic apoptotic pathway, we assessed the ratio of pro-apoptotic Bax to anti-apoptotic Bcl-2 proteins as a major checkpoint in the intrinsic apoptotic pathway, and the retinal levels of cytochrome c, which acts as a primary trigger of the apoptotic caspase cascade. As shown in Figure 7B, the Bax/Bcl-2 ratio increased by about 300% in MCE untreated mice as compared with controls. Niacin or citicoline at low dose did not influence the MCE-induced increase in Bax/Bcl-2 ratio, while at high dose, the ratio was reduced to about 50%. The combined administration of niacin with citicoline at low dose, further reduced the Bax/Bcl-2 ratio, reaching levels comparable to those in controls. Conversely, the Bax/Bcl-2 ratio did not significantly differ from that of MCE untreated mice after niacin combined with citicoline at high dose. The same trend of variations applies to the efficacy of the single components or their combined administration on protein levels of cytochrome c with higher efficacy of citicoline at low dose in combination with niacin at either low or high dose (Figure 7C).

## 4. Discussion

Increased intraocular pressure (IOP) is the main risk factor for developing glaucoma that is characterized by progressive optic nerve degeneration resulting in RGC death. Oxidative stress and inflammation work together to trigger apoptotic cell death by affecting mitochondrial dynamics. IOP elevation, characteristic of hypertensive glaucoma, was mimicked here by injecting the anterior chamber of the mouse eye with MCE, which clogs the trabecular meshwork and impairs the aqueous humor outflow. Sudden elevation of the IOP remains stable for almost 2 weeks, thus closely simulating the human hypertensive glaucoma. As shown by the present findings, a novel combination of niacin with citicoline has better efficacy over each single component in preserving RGC health in response to IOP increase by reducing inflammation and oxidative stress, which are the main triggers of RGC apoptotic death and retinal dysfunctional activity.

### 4.1. Characterization of the MCE model

Mechanic stress at the optic nerve head impairs RGC survival by activating multifactorial mechanisms among which the inflammatory cascade associated to glial cell activation triggers NF-κB, a transcriptional factor which enters the nucleus to generate high levels of pro-inflammatory cytokines, also including IL-6 (35). The inflammatory response is closely related to increased oxidative stress by enhancing the production of oxidative metabolites through the overexpression of ROS-producing enzymes (36). On its hand, increased oxidative stress, as determined by upregulated levels of the ROS-sensitive transcriptional factor Nrf-2 and its target HO-1, promotes an intracellular signaling cascade that activates a variety of transcription factors leading to enhanced pro-inflammatory gene expression (37). Upstream the IOP-associated oxidative stress, elevated mechanic stress and insufficient retinal perfusion impair mitochondrial biogenesis by affecting the activity of the electron transport chain, thus contributing to increased production of ROS finally leading to mitochondrial dysfunction and DNA alterations, eventually triggering the apoptotic cascade (38). Accordingly, we found that MCE injection leads to increased Bax/Bcl-2 ratio resulting in cytochrome c release as demonstrated by its increased expression. Then, activation of the apoptosis effector caspase-3 leads to a drastic decrease in immunostaining with RBPMS, a well-established marker of RGCs (39). Decreased density of RGCs is accompanied by impaired electroretinographic recordings of their activity. In fact, PhNR, a slow negative component of the photopic ERG that provides specific information about RGC activity, and PERG, an electrophysiologic response to a pattern reversing stimulus that objectively measures RGC function, are both reduced in amplitude in line with previous findings from different animal models of glaucoma with spontaneous or induced ocular hypertension (29, 30, 33, 40–43). Alterations in PhNR and PERG are also typical of glaucoma patients in which they have been possibly related to early RGC loss (9).

### 4.2. Efficacy of diet supplements

The MCE model used in this study has been previously employed to assess the efficacy of treatment strategies aimed at counteracting glaucomatous RGC degeneration by acting on IOP elevation or downstream inflammatory and oxidative processes. For instance, oral administration of nutritional products based on antioxidant/anti-inflammatory components effectively counteract MCE-induced RGC degeneration independently on IOP lowering (29) at variance with topically administered melatoninergic compounds, which exert a strong hypotensive effect (30). As shown by the present results, non-IOP-related mechanisms mediate the protective action of dietary supplementation with niacin and citicoline at their best dosage either alone or in combination. In particular, single administration of niacin or citicoline at doses in line with those previously reported to exert beneficial effects in preclinical models (21, 33), efficiently counteracts the major pathological signs of glaucoma at morpho/functional and molecular levels. Niacin supplementation would act by replacing the glaucoma-associated depletion of NAD^+^, a coenzyme involved in oxidative phosphorylation leading to ATP production. In fact, niacin has been shown to influence mitochondrial metabolism and its supplementation has been demonstrated to improve mitochondrial structure with the formation of more tightly folded cristae thus increasing the ATP-generating surface area (44, 45). On the other hand, citicoline exerts broader effects by elevating neurotrophin levels, ameliorating axonal transport deficits, restoring membrane integrity and improving mitochondrial function including cardiolipin synthesis that participates to ATP production (46).

### 4.3. Combined administration of niacin with citicoline

As also shown by the present results, the combined administration of niacin and citicoline at calibrated amounts is more effective than each single ingredient confirming the rationale for their association to limit retinal damage. This finding is in line with previous results demonstrating that the co-administration of vitamin B3 and citicoline also in combination with coenzyme Q10 is generally more effective than the single ingredients in reducing oxidation and inflammation (47). Combined efficacy of citicoline with CoQ10 has been demonstrated in many retinal pathologies including glaucoma (8). Accordingly, combined drug treatments acting by multitarget approach have been shown to be more effective compared with single-drug treatments (48). In combination, drugs could be used at doses lower than standard, thus displaying safer and more efficient activity. In the case of the MCE model of glaucoma used here, assuming that mitochondrial dysfunction mainly contributes to glaucoma-associated retinal damage and considering that mitochondrial cristae are the main target of vitamin B3, the obtained results demonstrate that the concomitant administration of niacin and citicoline has the theoretical advantage of better recovering mitochondrial health. However, the possibility that the combined efficacy of niacin and citicoline would not be limited to the improvement of mitochondrial health cannot be excluded given the broad spectrum of effects of citicoline, other than those specifically involving mitochondrial function. For instance, citicoline, acting as a source of phosphatidylcholine, has been shown to prevent neuronal membrane breakdown and apoptosis, thus providing neuroprotection (7). Moreover, citicoline may act as a choline donor for the synthesis of neurotransmitters such as acetylcholine (46). Given the evidence of the protective efficacy of acetylcholine receptor agonists on IOP-dependent RGC degeneration (49), the possibility exists that the neuroprotective effect of citicoline might also depend on enhanced cholinergic signaling.

**Figure 8:**
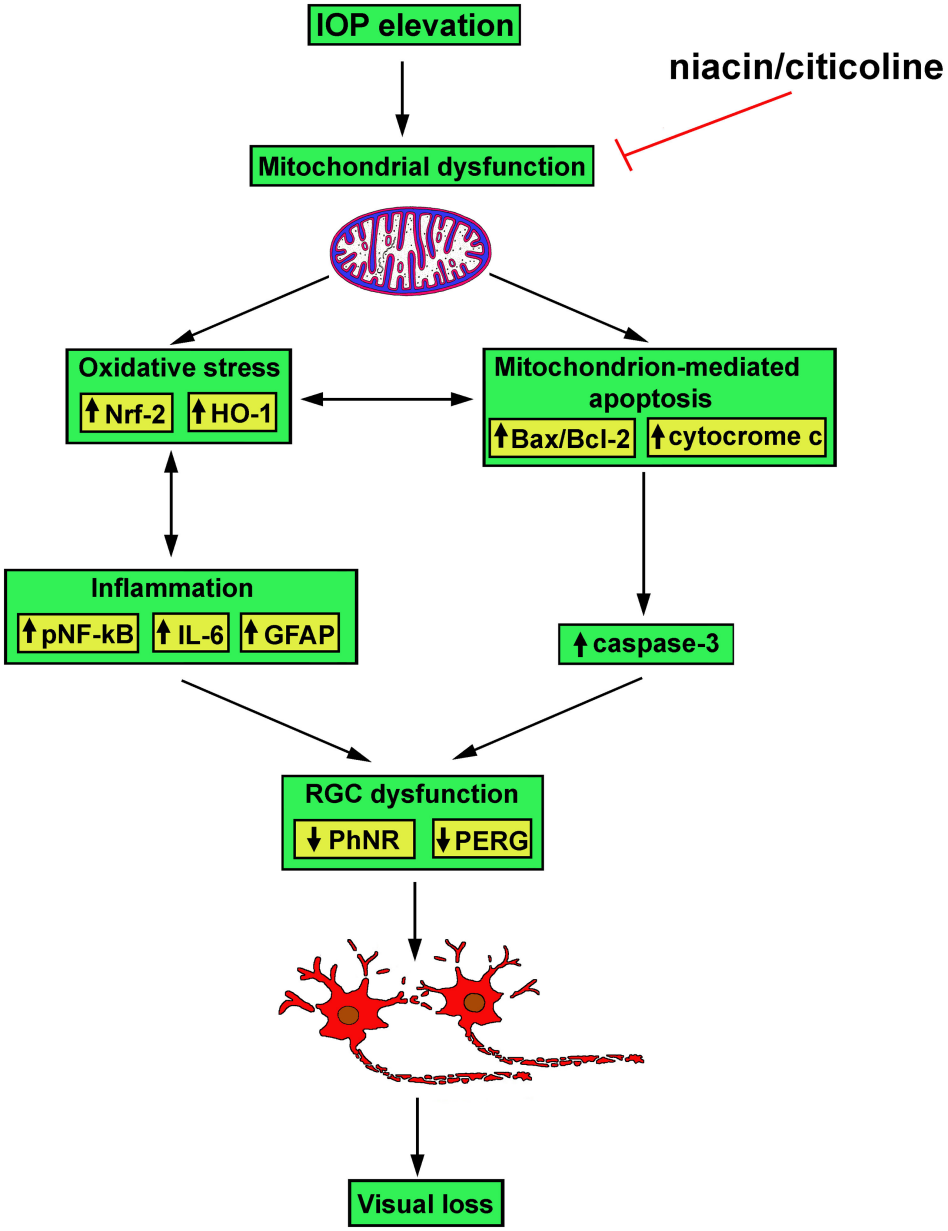
Schematic diagram depicting possible mechanisms of action of niacin and citicoline on hypertensive glaucoma. Niacin/citicoline, by intervening on dysfunctional mitochondria, would reduce oxidative stress and inflammation both resulting in preserved apoptotic cascade leading to retinal ganglion cell survival thus limiting visual loss.

As also shown by the present results, the efficacy of the combined administration of niacin and citicoline depends on their relative concentration, with best efficacy of each of them at high doses when administered alone, and even better efficacy when citicoline is administered at low dose in combination with niacin at either high or low dose. Combination of niacin with citicoline at their optimal ratio consistently acts on the different players in the molecular cascade triggered by IOP elevation towards RGC morpho-functional rescue (see the schematic diagram in Figure 8). Whether the combination efficacy depends on its preventive administration or rather reflects restored RGC health secondary to IOP injury remains to be answered although evidence is accumulating about lower-risk and high-benefit of dietary supplements in preventive care in respect to curative intervention (50).

The protective effects of niacin and citicoline are directly proportional to their concentration when given alone. Such effects can be improved by the association of either doses of niacin despite only in combination with low-dose citicoline. This finding is in line with the concept that low doses of two compounds that have different modes of action may provide additive effects to achieve better efficacy than high doses alone. On the other hand, the additive effect of the combination may vary according to the doses and the different proportions of each component in the combination (51). In the case of citicoline, previous evidence from a rat model of embolic stroke demonstrated that a suboptimal dose of citicoline combined with a thrombolytic therapy provided an additive efficacy on the reduction in infarct volume while increasing the dose of citicoline in the combination was less effective than its low dose (52) suggesting some kind of negative interference. We are currently unable to provide a clear explanation about the lack of combined efficacy when citicoline is given at high dose, but we can speculate about its reason. It is usually assumed that ingested citicoline is quickly hydrolyzed into cytidine and choline of which choline concentration in the plasma is much lower than cytidine thus preventing cholinergic toxicity (53). When high-dose citicoline is given in combination with niacin then choline concentration might exceed its physiological level as nicotinamide potentiates choline availability by blocking its clearance (54). Additional choline availability might derive from niacin interaction with activated phospholipase A2, which releases choline from choline-containing phospholipids (55). The final effect would be an intracellular choline accumulation beyond the level of toxicity (56). This would counterbalance the protective effect of each molecule either alone or in combination at low doses to ultimately drive apoptotic RGC death. Additionally, citicoline transport may be limited by high extracellular choline concentration with higher uptake maintained in the presence of low compared with high substrate level. Vitamin B3 diffusion, in contrast, involves high-affinity carrier-mediated mechanisms that render its transport less dependent on the substrate concentration.

### 4.4. Conclusion

Combination efficacy of niacin with citicoline is founded on multifactorial mechanisms of action that lead to improved mitochondrial resilience against glaucomatous stress. While drug combination is extensively studied for increasing therapy efficacy, preventing drug resistance and reducing therapy duration, less information is available on combined activity of distinct dietary supplements. In fact, supplement interactions are difficult to interpret because the variability in supplement constituents, quality, and dosage makes it difficult to dissect the efficacy of each component in respect to supplement combination.

Although the present findings suggest a potential effect of the combination in the experimental mouse model of hypertensive glaucoma, these results need to be further elucidated and reproduced in humans mostly in respect to the short follow up period in the MCE model. In this respect, the gradual increase in IOP with age associated to progressive loss of RGCs in the DBA/2J inbred mouse strain would allow us to follow for a longer time the temporal profile of RGC loss and IOP elevation (57). Additional restrictions apply on the daily doses allowed in humans that are much lower in respect to those used in animal models. Considering the different metabolism and size of a mouse, the doses used in this study are 50 to almost 260 times higher for niacin and 5 to 10 times higher for citicoline. This is justified by the fact that the insurgence of glaucoma and RGC apoptotic death occurs within 2 weeks in the mouse model as compared to decades in humans, thus much higher doses of the supplements are necessary to demonstrate their efficacy. In addition, there are many persistent gaps in our knowledge of supplement transport from the gut to the bloodstream and to the eventual target tissues in the eye. Further limitations include the difficulty to conduct large clinical trials using diet supplements to assess their impact on human health. In the case of vitamin B3, for instance, short-term clinical studies in glaucoma patients have demonstrated its efficacy in improving visual function, but additional clinical trials with longer follow-up are needed before vitamin B3 may be incorporated into clinical practice. In the case of niacin combination with citicoline, the present findings are highly suggestive of the possibility that it may become part of the adjuvant treatment of glaucoma although its therapeutic potential needs to be further explored with both preclinical and randomized clinical trials.

## Data availability statement

The raw data supporting the conclusions of this article will be made available by the authors, without undue reservation.

## Ethics statement

The animal study was reviewed and approved by Commission for Animal Wellbeing of the University of Pisa.

## Author contributions

DR, PB, and MC: conceptualization and project administration. RA and AM: methodology, validation, and investigation. RA, AM, and MC: formal analysis and data curation. MC: resources, supervision, and funding acquisition. PB and MC: writing–original draft preparation. RA, MD, DR, PB, and MC: writing–review and editing. All authors contributed to the article and approved the submitted version.

## Funding

This study was supported by funding from the Italian Ministry of Universities and Research to MC (FRA-2021/2022) and RA (FRA-2023). This study was also supported by the Italian Ministry of Universities and Research under the Department of Excellence 2023–2027 initiative. This research was also funded by the financial support of Fidia Farmaceutici S.p.A. Abano Terme, Italy.

## Conflict of interest

DR was an employee of Fidia Farmaceutici S.p.A.

The remaining authors declare that the research was conducted in the absence of any commercial or financial relationships that could be construed as a potential conflict of interest.

This study received funding from Fidia Farmaceutici S.p.A. The funder had the following involvement with the study: decision to publish the results.

## Publisher’s note

All claims expressed in this article are solely those of the authors and do not necessarily represent those of their affiliated organizations, or those of the publisher, the editors and the reviewers. Any product that may be evaluated in this article, or claim that may be made by its manufacturer, is not guaranteed or endorsed by the publisher.
